# Correlation of Arterial Lactate and pH With the Immediate Outcome of Term Asphyxiated Neonates

**DOI:** 10.7759/cureus.80093

**Published:** 2025-03-05

**Authors:** Rayesa Authifa, Poorva Gohiya, Jyotsna Shrivastava

**Affiliations:** 1 Pediatrics, Gandhi Medical College Bhopal, Bhopal, IND

**Keywords:** biomarkers, hearing loss, neonatal mortality, neurological outcomes, perinatal asphyxia, ph, serum lactate

## Abstract

Introduction: Perinatal asphyxia is a significant cause of neonatal morbidity and mortality. Early identification of neonates at risk for adverse outcomes is crucial for timely intervention. This study aimed to evaluate the prognostic value of arterial lactate and pH levels in predicting immediate clinical outcomes in term asphyxiated neonates. Additionally, the study explored the potential association between elevated lactate levels and hearing impairment.

Methods: This observational cross-sectional study was conducted at a tertiary care center in Bhopal, India. A total of 100 term neonates with birth asphyxia were enrolled. Neonates admitted within six hours of birth had their arterial lactate and pH levels measured within the first hour of neonatal intensive care unit (NICU) admission. Survival rates, neurological outcomes at discharge, and hearing assessment of asphyxiated neonates through otoacoustic emissions (OAE) were the primary and secondary objectives. Statistical analysis involved Pearson’s correlation and receiver operating characteristic (ROC) curves to determine optimal pH and lactate thresholds for predicting adverse outcomes.

Results: A lactate threshold of 6.2 mmol/L was predictive of neurological abnormalities (area under the curve (AUC) 0.85, sensitivity 76%, specificity 79%), while a threshold of 10 mmol/L was associated with increased mortality (AUC 0.77, sensitivity 59%, specificity 88%). pH levels <7.2 were linked to a higher risk of death (AUC 0.79, sensitivity 71%, specificity 77%). pH levels below 7.3 were associated with a greater risk of neurologically abnormal neonates (AUC 0.78, sensitivity 83%, specificity 62%). Neonates who failed OAE screening had significantly higher lactate levels compared to those who passed, suggesting an association between higher lactate values and hearing impairment in asphyxiated neonates. The median (IQR) lactate levels for the B/L Pass and B/L Refer groups were 4.3 (3.1, 6.8) and 7.4 (6.3, 10.3), respectively.

Conclusion: Arterial lactate and pH are valuable biomarkers for the early prediction of neurological abnormalities, mortality, and hearing impairment in term asphyxiated neonates. Integrating these measurements into neonatal care protocols may enhance risk stratification and guide early interventions.

## Introduction

Perinatal asphyxia, characterized by insufficient oxygenation and perfusion to the fetus or neonate, represents a predominant etiological factor in neonatal mortality, accounting for approximately 23% of global neonatal deaths [1]. Globally, there were 4.9 million under-five deaths in 2022, and 2.3 million occurred during the first month of life. Birth asphyxia accounts for almost 14% of under-five deaths. The neonatal mortality rate is 18 per 1,000 live births in India. The major causes of this mortality are prematurity, birth asphyxia, and congenital malformations [2].

However, the implications of neonatal asphyxia extend beyond immediate fatality; they encompass long-term neurodevelopmental defects and chronic health complications. Consequently, effective management is imperative for enhancing short- and long-term prognosis. Perinatal asphyxia causes a cascade of cellular events, including increased production of free radicals, mitochondrial dysfunction, anaerobic metabolism, and apoptosis. The pathophysiological mechanisms underlying neonatal asphyxia involve oxygen deprivation, which initiates anaerobic metabolic processes. This, in turn, culminates in an increase in lactate levels and a reduction in pH, indicative of metabolic acidosis. These biochemical markers are essential for evaluating the extent of hypoxia and informing clinical decision-making [3]. Several research endeavors have elucidated the relationship between umbilical cord lactate concentrations and neonatal outcomes, establishing it as a crucial prognostic metric within the clinical milieu [4-6].

Asphyxiated neonates frequently endure immediate clinical repercussions, which may manifest as multi-organ dysfunction involving the central nervous, cardiac, renal, pulmonary, and hematological systems. Neurological sequelae, such as seizures and hypoxic-ischemic encephalopathy (HIE), have the potential to precipitate enduring developmental obstacles. The brain damage caused by HIE is not uniform. The most vulnerable areas are the cerebral cortex, hippocampus, and cerebellum, which are responsible for higher cognitive functions, memory, and motor coordination [7]. The mortality rate for severely asphyxiated infants can reach alarming heights, approximately 30% [8]. The early detection of heightened lactate levels and reduced pH is instrumental in orchestrating timely interventions; such actions are vital for enhancing survival probabilities and mitigating long-term sequelae. Nonetheless, despite the acknowledged significance of lactate and pH in neonatal care, considerable deficiencies persist in comprehending how specific thresholds of these biomarkers can forecast distinct clinical outcomes. Although previous investigations have delineated broad correlations, the exact relationship between these biochemical indicators and outcomes such as neurological impairment and multi-organ dysfunction remains underexplored [9].

A known consequence of neonatal asphyxia is sensorineural hearing loss. Although this condition may not manifest at birth, undetected hearing loss can significantly affect a child’s long-term development. Early hearing assessments in asphyxiated neonates are critical for identifying and addressing potential defects. An understudied topic is understanding the correlation between elevated lactate levels and hearing impairment. It might enhance neonatal management by facilitating the early detection of at-risk infants. Given these clinical challenges, this study aims to evaluate the correlation between arterial lactate and pH concerning immediate clinical outcomes such as neurological function and mortality. Furthermore, this study intends to explore the association between elevated lactate and hearing impairment in term asphyxiated newborns. These findings will contribute to a more nuanced understanding of how biochemical markers can guide clinical decisions and improve neonatal care, thus enhancing patient outcomes.

## Materials and methods

This was a hospital-based, cross-sectional study conducted in the neonatal intensive care unit (NICU) of the Department of Pediatrics at a tertiary care teaching hospital. The study was carried out over a 13-month period, from October 2022 to October 2023. The study population consisted of term asphyxiated newborns referred to the NICU within six hours of birth. Term neonates (≥37 weeks’ gestation) diagnosed with birth asphyxia, defined as the need for continued resuscitation (positive pressure ventilation or assisted ventilation at birth) and clinical evidence of encephalopathy (e.g., seizures, hypotonia), and in whom asphyxia could not be attributed to any other cause were included. Newborns with lethal congenital anomalies or those who were not referred within six hours of life were excluded.

The sample size was calculated based on an estimated prevalence (p) of 7% for birth asphyxia among term neonates. This 7% was derived from hospital statistics over the past three years. The sample size was calculated using the formula:

$n=\frac{z&sup2;(p\times q)}{d&sup2;}$

where z is the confidence level (1.96 for 95%), p = 7, q = 100 − p = 93, and d = 5% (precision). The calculated sample size was 100 neonates. All eligible neonates admitted to the NICU and meeting the inclusion criteria during the study period were consecutively enrolled until the target sample size was reached.

The primary study tool was an ABG analyzer (GEM Premier 3500) for measuring arterial lactate, pH, and other relevant parameters, including base excess, bicarbonate, PO₂, and PCO₂. A heparinized syringe was used to collect arterial blood samples from the radial artery, which were immediately processed in the ABG analyzer to ensure accuracy. Calibration of the ABG analyzer was regularly performed per manufacturer guidelines, and a log for calibration was maintained daily. A standardized, pretested, semi-structured interview schedule was used to collect demographic, clinical, and laboratory details. For term asphyxiated neonates admitted within six hours of birth, arterial blood gas (ABG) analysis was performed within one hour of admission. The neonates were followed until discharge or death, and overall clinical outcomes were categorized as survival or death. Neurological outcomes were classified as either "neurologically normal" or "neurologically abnormal." Hearing was assessed using otoacoustic emissions (OAE) at discharge, with results categorized as either "bilateral pass" or "bilateral refer." The interview schedule and data collection tools were pretested on a small subset of patients (not included in the final study), and necessary adjustments were made based on feedback from the pretest. Data collection was carried out by a trained resident who underwent training on the study objectives and methodology to ensure uniformity. Training sessions covered the correct procedure for collecting arterial blood samples, operation of the ABG analyzer, and accurate completion of the interview schedule. Data entry was double-checked by a senior investigator to prevent transcription errors. The ABG analyzer was regularly calibrated, and duplicate measurements were taken on a random subset of samples to verify the accuracy of lactate and pH levels. The study followed a standardized protocol for monitoring neonates, ensuring the reliability of outcome measurements (neurological and hearing assessments).

Data privacy was strictly maintained throughout the study. The study protocol was reviewed and approved by the Institutional Ethics Committee. Informed written consent was obtained from the parents or guardians of all participating neonates after explaining the study’s purpose and procedures.

Data were entered using Microsoft Excel (Microsoft Corporation, Redmond, Washington) and analyzed using STATA version 14 (StataCorp LLC, College Station, Texas) and R software version 4.4.1 (R Foundation for Statistical Computing, Vienna, Austria). Descriptive statistics were used to summarize demographic and clinical characteristics. Continuous variables were presented as mean ± SD and medians with interquartile ranges. Pearson’s correlation was employed to assess the relationship between arterial lactate and pH values with immediate neonatal outcomes (survived vs. deceased). Receiver operating characteristic (ROC) curve analysis was performed to determine the predictive capacity of lactate and pH for both neurological and survival outcomes. The area under the curve (AUC) was calculated with a 95% confidence interval (CI). Sensitivity, specificity, and optimal cutoff points were calculated using the Youden Index. A p-value <0.05 was considered statistically significant.

## Results

During the study period, a total of 336 asphyxiated neonates were admitted to the NICU. Of these, 100 term asphyxiated neonates meeting the inclusion criteria were selected for analysis. Among the selected neonates, 61% were male, with a mean ± SD birth weight of 2.7 ± 0.5 kg, and 66% were outborn (referred) cases. The mean ± SD age at admission was 105.7 ± 84.7 minutes, and the mean ± SD time from admission to arterial blood gas (ABG) collection was 32.4 ± 9.1 minutes. The mean ± SD maternal age was 24.6 ± 3.7 years. Among the mothers, 93.3% had preeclampsia as a risk factor, all had adequate amniotic fluid, and 31% presented with meconium-stained amniotic fluid. During labor, 42% of the mothers experienced a prolonged second stage, and 15% had obstructed labor. Normal vaginal delivery occurred in 84% of cases, with the most common reason for cesarean section (LSCS) being fetal distress (31%), followed by a history of previous LSCS (12%).

None of the neonates cried immediately after birth, and bag-and-mask ventilation was the most frequently employed resuscitation technique, used in 68% of cases. At the time of admission, 68% of the neonates were alert, while 28% were lethargic. Additionally, 15% had an absent cry, 13% exhibited increased muscle tone, 22% were limp, 14% experienced convulsions, 31% lacked a sucking reflex, and 18% had a poor sucking reflex. In terms of responsiveness, 21% of the neonates responded to pain stimuli. The immediate outcomes included either discharge or death, with 83% of the neonates being discharged, while 17% did not survive. Furthermore, 42% of the neonates exhibited neurological abnormalities. Of the 83 neonates who underwent hearing assessments using OAE, 71 (85.5%) passed the hearing assessment, while the remainder required further evaluation. A detailed comparison of immediate outcomes is shown in Table 1.

**Table 1:** A comparison of demographic and clinical variables by immediate outcome (N=100) p value <0.05 is considered significant. Statistical tests used were chi-square test for categorical variables and an independent t-test for continuous variables. ABG: arterial blood gas, AVPU: alert, voice, pain, unresponsive, LSCS: lower segment cesarean section, NVD: normal vaginal delivery, OAE: otoacoustic emissions.

S.No	Variable	Category	Survived (n = 83)	Expired (n = 17)	p-value
1	Sex	Male	50 (82.0%)	11 (18.0%)	0.73
		Female	33 (84.6%)	6 (15.4%)	
2	Place of delivery	Inborn	29 (85.3%)	5 (14.7%)	0.66
		Out born	54 (81.8%)	12 (18.2%)	
3	Weight (kg)	-	2.7 (0.5)	2.7 (0.5)	0.75
4	Age at admission (minutes)	-	102.7 (86.5)	120.4 (76.5)	0.49
5	Time to ABG (minutes)	-	32.4 (9.2)	31.9 (9.1)	0.09
6	Maternal age (years)	-	24.9 (3.9)	23.4 (2.3)	0.14
7	Maternal risk factors	Pre-eclampsia	12 (85.7%)	2 (14.3%)	0.08
		Eclampsia	0 (0.0%)	1 (100.0%)	
		None	71 (83.5%)	14 (16.5%)	
8	Type of delivery	LSCS	14 (87.5%)	2 (12.5%)	0.60
		NVD	69 (82.1%)	15 (17.9%)	
9	Resuscitation methods	Tactile stimulation	1 (100.0%)	0	0.06
		Only oxygen	17 (89.5%)	2 (10.5%)	
		Bag and mask	59 (86.8%)	9 (13.2%)	
		Intubation	6 (54.5%)	5 (45.5%)	
		Chest compression	0 (0.0%)	1 (100.0%)	
10	Tone	Active	64 (98.5%)	1 (1.5%)	<0.01
		Limp	9 (40.9%)	13 (59.1%)	
		Increased tone	10 (76.9%)	3 (23.1%)	
11	Convulsion	None	68 (82.9%)	14 (17.1%)	0.88
		Present on admission	12 (85.7%)	2 (14.3%)	
		Past history	3 (75.0%)	1 (25.0%)	
12	Sucking	Absent	18 (58.1%)	13 (41.9%)	<0.01
		Good	50 (98.0%)	1 (2.0%)	
		Poor	15 (83.3%)	3 (16.7%)	
13	AVPU	Alert	70 (94.6%)	4 (5.4%)	<0.01
		Unconscious	0 (0.0%)	4 (100.0%)	
		Pain	12 (57.1%)	9 (42.9%)	
		Vocal	1 (100.0%)	0 (0.0%)	
14	pH	-	7.3 (0.1)	7.2 (0.2)	<0.01
15	Lactate (mmol/L)	-	5.7 (3.2)	9.2 (3.5)	<0.01
16	Bicarbonate (mmol/L)	-	14.6 (3.8)	10.3 (5.2)	<0.01
17	Base Excess (mmol/L)	-	-10.3 (4.5)	-15.2 (6.2)	<0.01
18	Neurological status	Abnormal	25 (59.5%)	17 (40.5%)	<0.01
		Normal	58 (100.0%)	0 (0.0%)	
19	Hearing assessment (OAE)	Bilateral pass	71 (100.0%)	0 (0.0%)	-
		Bilateral refer	12 (100.0%)	0 (0.0%)	

**Figure 1:**
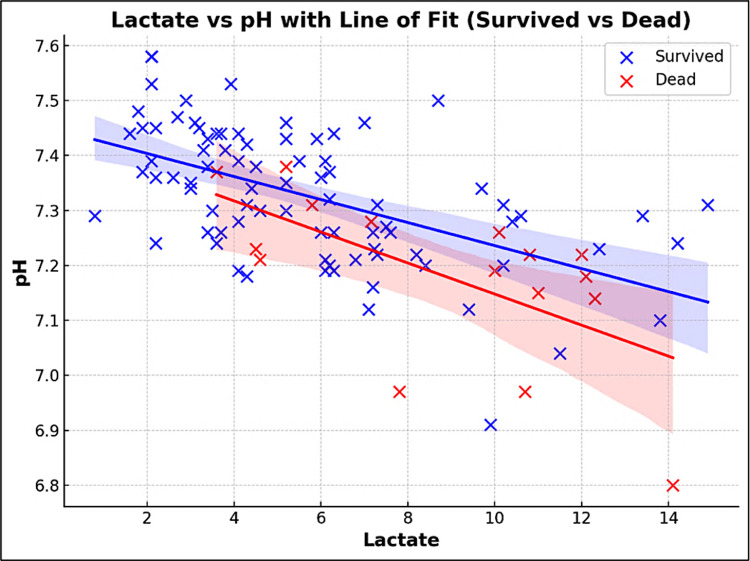
A scatter plot showing the correlation levels between lactate levels and pH in terms of outcome (Survival vs Dead) Pearson's correlation test was used.

The correlation analysis between arterial pH and lactate levels revealed a moderately strong negative correlation, with a Pearson correlation coefficient of -0.64 and it is statistically significant, with a p-value of <0.01 as shown in Figure 1.

Lactate level and hearing impairment

**Figure 2:**
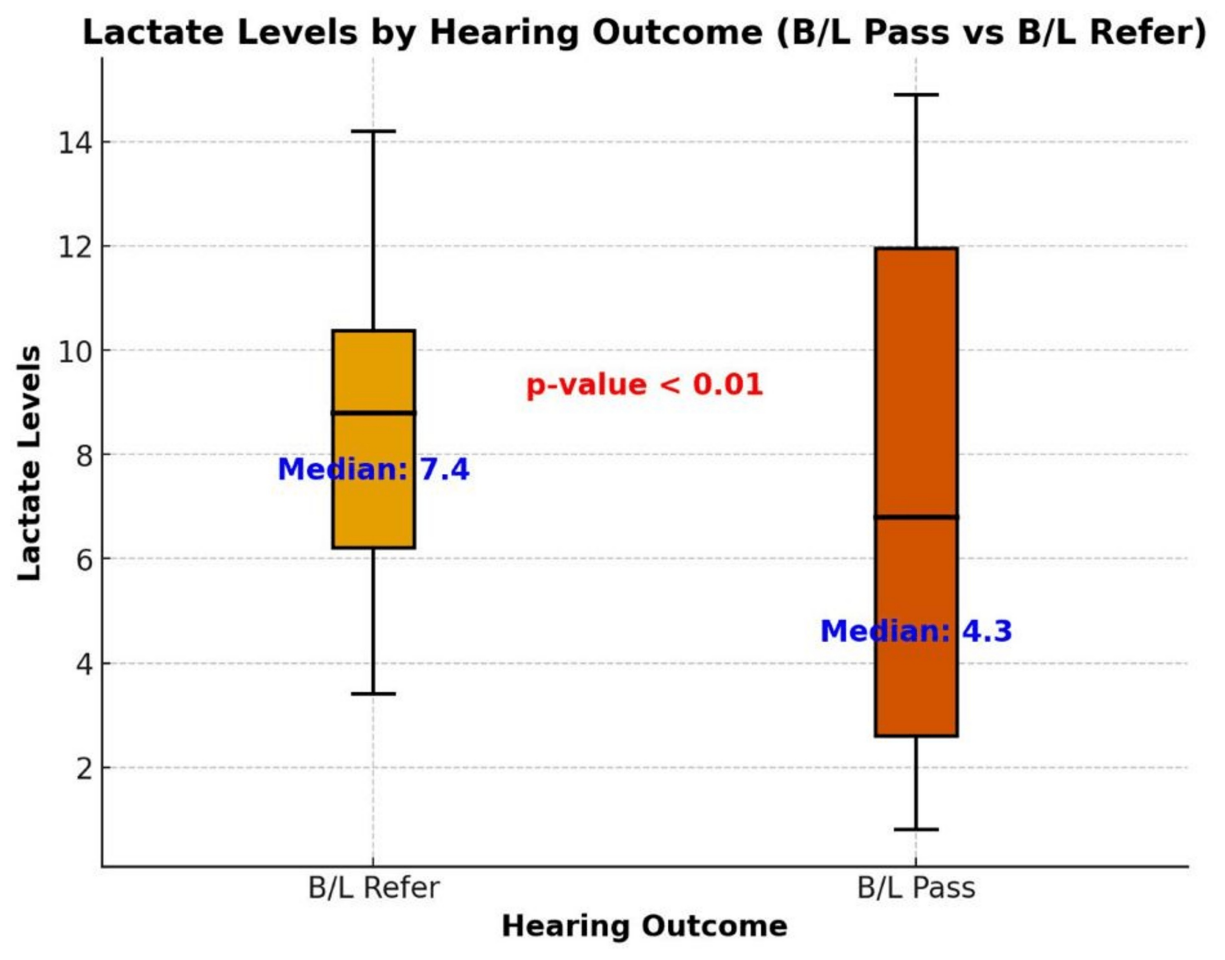
A comparison of lactate levels based on hearing outcome: bilateral pass vs bilateral refer Box and Whisker plot. p-value <0.05 is considered significant.

Figure 2 illustrates the distribution of lactate levels in neonates based on their hearing outcomes, comparing those who passed (B/L Pass) versus those who failed (B/L Refer). The median (IQR) lactate levels for the B/L Pass and B/L Refer groups are 4.3 (3.1, 6.8) and 7.4 (6.3, 10.3), respectively. A significant difference is observed between the two groups, with a p-value of <0.01, indicating that lactate levels are significantly associated with hearing outcomes in asphyxiated neonates.

Lactate level and immediate outcome

**Figure 3:**
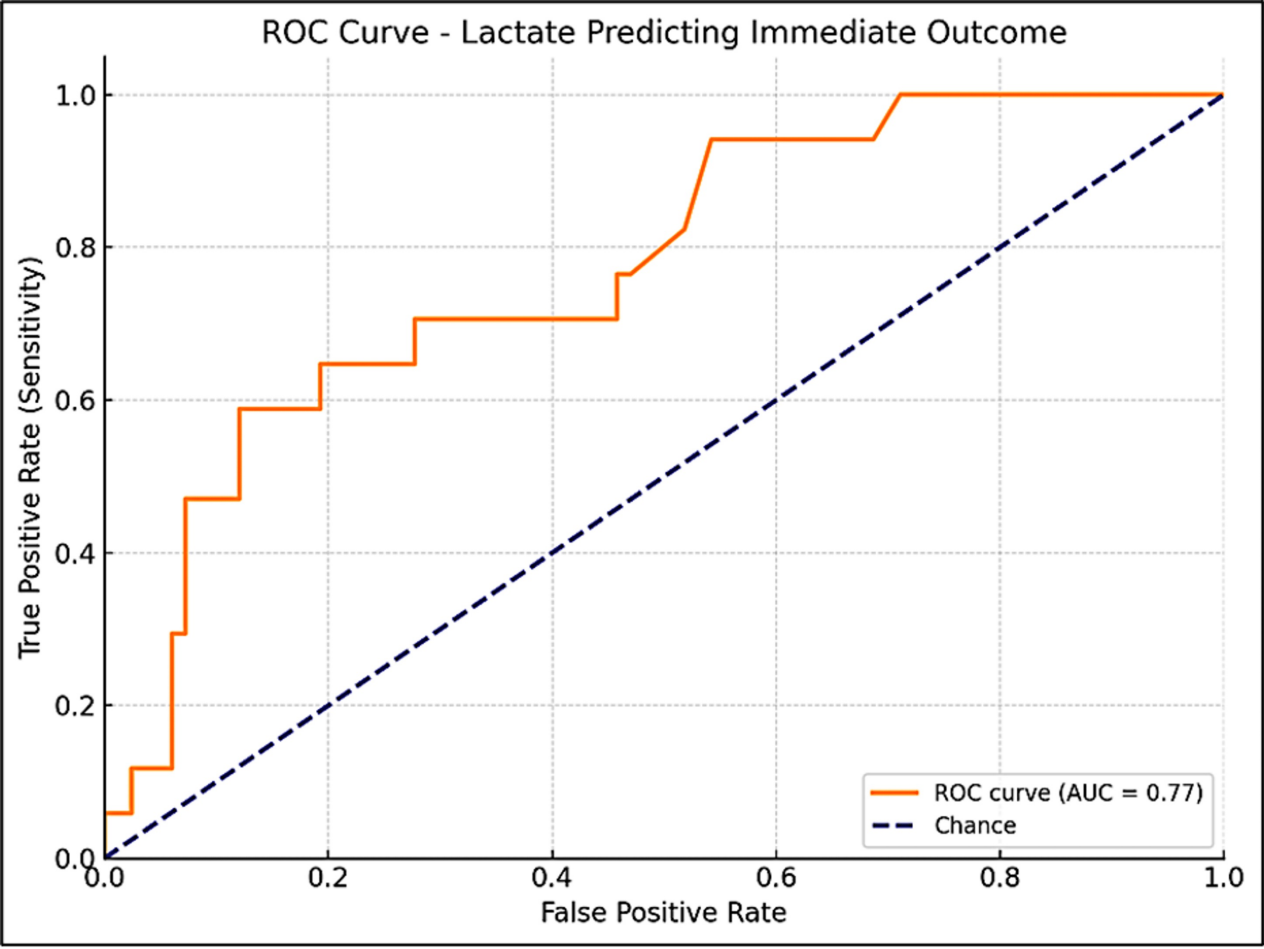
ROC curve for arterial lactate in predicting immediate survival outcome in term asphyxiated neonates ROC, receiver operator curve. The area under the curve was calculated with a 95% confidence interval (CI).

**Figure 4:**
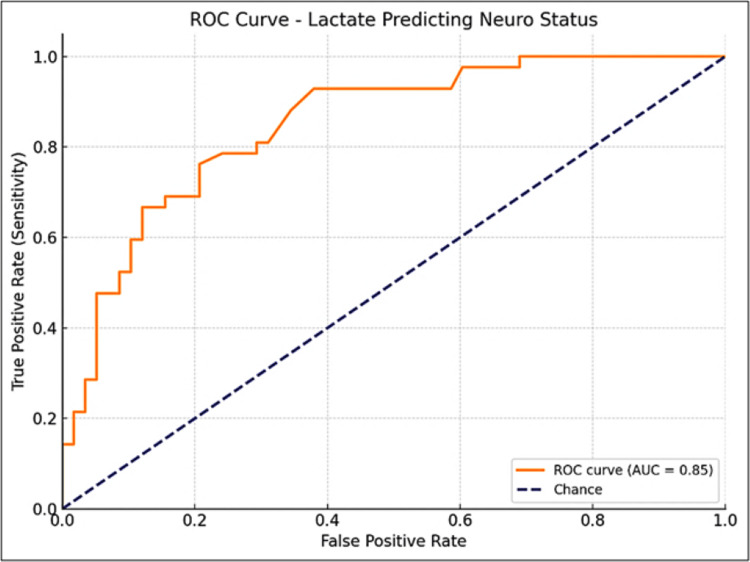
ROC curve for arterial lactate in predicting neurological outcome in term asphyxiated neonates ROC, receiver operator curve. The area under the curve was calculated with a 95% confidence interval (CI).

The ROC analysis for lactate levels in predicting immediate outcomes in term asphyxiated neonates identified 10 mmol/L as the optimal cutoff, with a Youden Index of 0.47 and an AUC of 0.77 (95% CI: 0.64-0.88). At this threshold, the sensitivity is 59% and the specificity is 88%, indicating that lactate levels ≥10 mmol/L are strongly associated with a higher risk of death (Figure 3). For predicting neurological outcomes, a lactate cutoff of 6.2 mmol/L was identified, with a Youden Index of 0.56 and an AUC of 0.85 (95% CI: 0.64-0.88). This threshold had a sensitivity of 76% and a specificity of 79%, indicating a strong association with neurologically abnormal neonates (Figure 4).

pH level and immediate outcome

**Figure 5:**
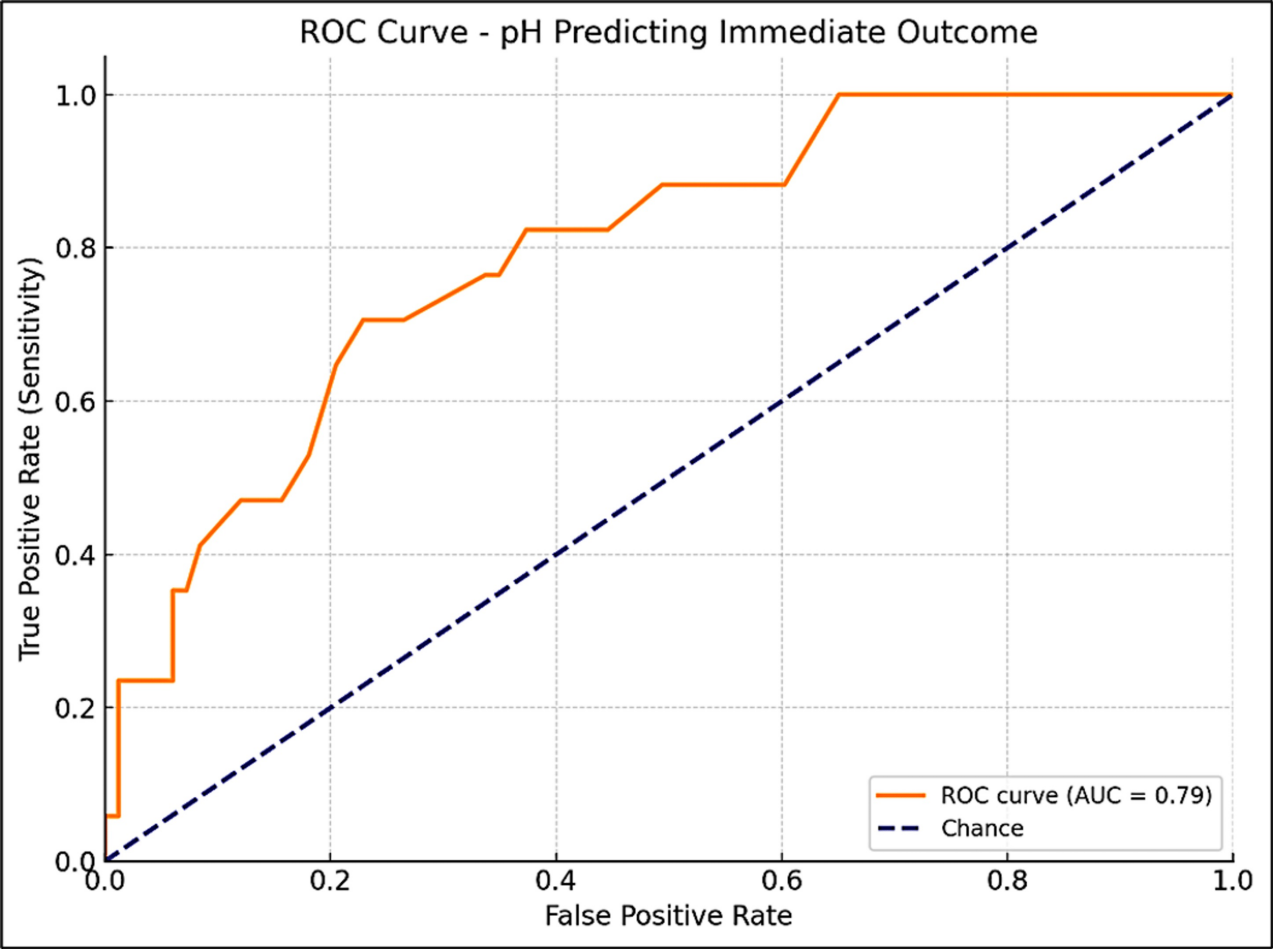
ROC curve for arterial pH in predicting immediate survival outcome in term asphyxiated neonates ROC, receiver operator curve. The area under the curve was calculated with a 95% confidence interval (CI).

**Figure 6:**
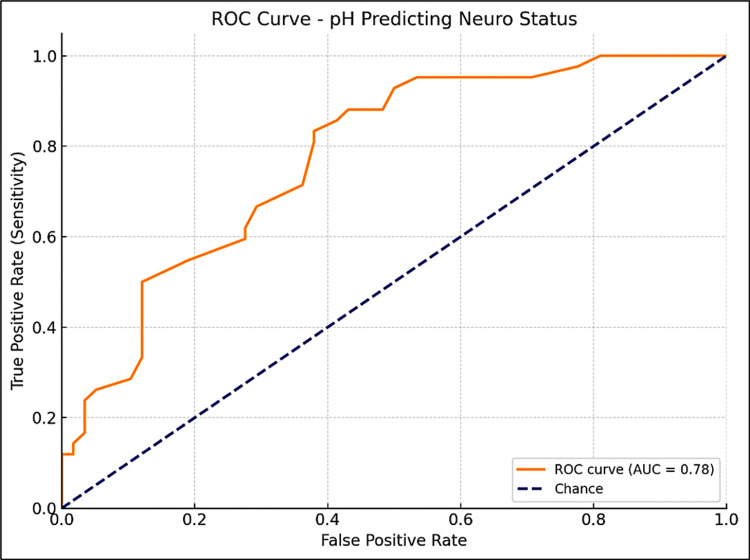
ROC curve for arterial blood pH in predicting neurological outcome in term asphyxiated neonates ROC, receiver operator curve. The area under the curve was calculated with a 95% confidence interval.

Similarly, pH levels were analyzed. For predicting immediate outcomes, a pH cutoff of 7.2 was determined, with a Youden Index of 0.48 and an AUC of 0.79 (95% CI: 0.68-0.91), showing 71% sensitivity and 77% specificity. A pH of less than 7.2 was associated with a higher risk of adverse outcomes (Figure 5). For neurological outcomes, a pH cutoff of 7.3 was identified, with a Youden Index of 0.45 and an AUC of 0.78 (95% CI: 0.68-0.91), yielding 83% sensitivity and 62% specificity, suggesting that pH levels below 7.3 are linked to a greater risk of neurological abnormalities (Figure 6).

## Discussion

Our study aimed to analyze the correlation between arterial lactate and pH with immediate clinical outcomes in term asphyxiated neonates, while also exploring the occurrence of hearing impairment in relation to lactate levels. The key findings revealed a significant inverse correlation between arterial lactate and pH levels, reinforcing their combined utility in predicting neonatal outcomes. Lactate levels ≥10 mmol/L were found to be a strong predictor of mortality (AUC: 0.77, 59% sensitivity, 88% specificity), while a lactate cutoff of 6.2 mmol/L was associated with neurological abnormalities (AUC: 0.85, 76% sensitivity, 79% specificity). pH <7.2 was linked with increased mortality, and pH <7.3 was associated with a higher likelihood of neurological damage. These results underscore the importance of both lactate and pH as biomarkers for early risk stratification in neonates suffering from perinatal asphyxia.

The findings of our study align with a growing body of literature that emphasizes the prognostic value of lactate levels and pH in predicting mortality and neurological outcomes in asphyxiated neonates. A systematic review by Matsushita et al. [9] highlighted the association between elevated lactate concentrations and increased mortality rates in neonates, underscoring lactate’s role as a reliable biomarker for adverse outcomes. Similarly, Van et al. [10] reported that umbilical cord lactate levels are significantly associated with the presence and severity of HIE, further reinforcing the need for early lactate assessment in predicting neurological outcomes. Several studies further validate lactate’s prognostic value. Tuuli et al. [11] demonstrated that lactate is a more discriminating measure of neonatal morbidity than pH, with their large prospective cohort study identifying higher sensitivity and specificity for lactate (83.9% and 74.1%) compared to pH (75.0% and 70.6%). Qamar et al. [12] similarly found that lactate had the highest AUC for predicting NICU admissions and low Apgar scores. Neacsu et al. [13] also supported these findings by showing that lactate had a higher ROC curve area (0.92) compared to pH (0.75), reinforcing our conclusion that lactate is a more stable and reliable biomarker of metabolic distress and hypoxia.

Our study is also in agreement with Shah et al. [14], who showed that initial postnatal lactate levels are crucial predictors of short-term adverse outcomes in neonates with intrapartum asphyxia, highlighting the utility of lactate in guiding early interventions. Fernandez et al. [15] corroborated these findings by showing that plasma lactate concentrations greater than 4.2 mmol/L within the first six hours of life were associated with increased neonatal mortality and neurological morbidity, particularly in newborns small for their gestational age. While their threshold differs slightly from ours, both studies underscore the role of lactate as a reliable biomarker for predicting adverse neonatal outcomes. Moreover, Cheung et al. [16] demonstrated that plasma lactate levels are valuable for predicting early mortality and adverse outcomes in neonates undergoing extracorporeal membrane oxygenation (ECMO), with similar lactate thresholds for mortality and neurological outcomes as in our study. Simovic et al. [17] found that lactate levels exceeding 9.95 mmol/L predicted death with 75% sensitivity and 74.3% specificity, further supporting lactate as a critical early biomarker for severe outcomes.

In a study conducted by Kumar et al. [18], cord blood pH values below 7.2 were associated with higher rates of neonatal resuscitation (44.6%), NICU admissions (67.6%), early neonatal complications (53.4%), and neonatal death (10.1%). Bhat et al. [19] also reported a similar pH level of around 7.1 in neonates with HIE, which decreased with increasing severity of the condition. The pH of arterial blood is a critical marker of neonatal health and strongly correlates with neonatal mortality and morbidity. Studies show that a pH below 7.2 is linked to immediate complications such as low Apgar scores, the need for resuscitation, and NICU admission, as well as the development of HIE and cerebral palsy. Research by Eltaieb et al. [20], Martí Gamboa et al. [21], and Allanson et al. [22] supports the use of umbilical cord pH as a predictor of adverse outcomes.

The superiority of lactate over pH as a prognostic biomarker can be attributed to the physiological mechanisms involved in neonatal hypoxia. Lactate, a product of anaerobic metabolism, accumulates when oxygen supply to tissues becomes insufficient. Elevated lactate levels provide a direct reflection of the severity of hypoxia and the extent of tissue damage. In contrast, pH can fluctuate due to various compensatory mechanisms, such as the administration of bicarbonate during resuscitation, which may temporarily stabilize pH without resolving the underlying hypoxia [23,24]. Lactate’s more consistent rise in response to metabolic distress makes it a more reliable and stable biomarker for assessing the severity of hypoxic injury. This is particularly important in guiding clinical interventions, such as therapeutic hypothermia, in neonatal care [25].

Given the established link between perinatal asphyxia and auditory impairments, our findings extend the clinical utility of lactate as a biomarker for multiple organ dysfunctions. In our cohort, 14.5% of neonates failed the OAE hearing screening, and these neonates exhibited significantly higher lactate levels, reinforcing the connection between severe asphyxia, elevated lactate, and hearing impairment. This aligns with the findings of Pawar et al. [26], Riyaz et al. [27], and Bharathi MB et al. [28], who reported higher rates of hearing impairment in neonates with severe asphyxia. Our study provides additional evidence that lactate, beyond predicting neurological outcomes, may serve as a biomarker for auditory impairments, a novel contribution to the existing literature.

Strength and limitations

This study is among the few that concurrently examine both arterial lactate and pH as biomarkers for predicting neurological and hearing outcomes in term asphyxiated neonates, offering a more comprehensive framework for risk assessment. The identification of specific lactate thresholds provides actionable insights, potentially guiding early clinical interventions and improving neonatal outcomes. Notably, our study uniquely explores the association between elevated lactate levels and hearing impairment, a less-explored area in neonatal care. However, this study has certain limitations. It is an observational study and a single-center study, which may affect the generalizability of the findings.

## Conclusions

The study demonstrates that elevated arterial lactate levels and lower pH are significant predictors of adverse neurological outcomes, mortality, and hearing impairment in term asphyxiated neonates. A lactate threshold of 6.2 mmol/L strongly correlates with neurological abnormalities, while a threshold of 10 mmol/L is associated with increased mortality. Similarly, a pH threshold of <7.2 is linked to a higher risk of death, and a pH of <7.3 predicts greater neurological risk. Additionally, lactate levels were predictive of hearing impairment, providing novel insight into its broader clinical utility. These findings underscore the importance of early lactate and pH measurement in guiding timely interventions and improving prognosis in neonates suffering from perinatal asphyxia. Further research with larger cohorts and standardized protocols is necessary to validate these findings and refine the clinical application of lactate and pH as biomarkers.
